# REFA‐DINO: A Relative‐Efficient Fusion Attention Framework for Binary and Multiclass Diabetic Retinopathy Classification

**DOI:** 10.1155/ijbi/4374759

**Published:** 2026-08-25

**Authors:** Hussain Dawood, Muniba Noreen, Ali Javed, Hafsa Ilyas, Usman Muhammad, Hassan Dawood

**Affiliations:** ^1^ International Center for Materials Sciences and Technology, Western Caspian University, Baku, Azerbaijan, wcu.edu.az; ^2^ Jadara Research Center, Jadara University, Irbid, Jordan, jadara.edu.jo; ^3^ Department of Software Engineering, University of Engineering and Technology, Taxila, Taxila, Pakistan, vnu.edu.vn; ^4^ Department of Computer Science, Aalto University, Espoo, Finland, aalto.fi

**Keywords:** computer-aided diagnosis, diabetic retinopathy, DINOv2, EfficientNet, medical imaging classification, vision transformers

## Abstract

Diabetic retinopathy (DR) has become a major cause of preventable blindness, and its early detection is crucial for maintaining vision in diabetic patients. Existing computer‐aided diagnosis (CAD) systems still face challenges in detecting early‐stage lesions, integrating local and global features, and maintaining consistent performance in binary and multiclass settings. To address these limitations, this study proposes a relative‐efficient fusion attention framework (REFA‐DINO) that integrates a convolutional local‐feature learning module with a transformer‐based global contextual method. The architecture employs a relative‐efficient fusion adapter (REFA) block comprising two branches, EfficientNet and attention‐enhanced patch embedding. EfficientNet extracts lesion‐sensitive retinal features, and an attention‐enhanced patch‐embedding module is used to generate token representations. An adaptive relative position‐aware cross‐attention mechanism is introduced to enable effective interaction among heterogeneous features, which are further processed through the DINO‐based transformer module. The extracted global representations are passed to the linear classification head for DR classification. REFA‐DINO is evaluated on two diverse and large‐scale fundus imaging datasets, EyePACS and APTOS, for binary and multiclass classification. The proposed framework achieved accuracies of 98.60% and 93.77% for binary classification and 85.52% and 87.51% for multiclass classification on the APTOS and EyePACS datasets, respectively. A comparative analysis against state‐of‐the‐art methods, cross‐corpora evaluation, and an ablation study are also conducted to demonstrate the effectiveness of the proposed REFA‐DINO. Compared with state‐of‐the‐art methods, the REFA‐DINO framework enhances binary classification on both the EyePACS and APTOS datasets, with significant improvements over the strongest baselines. The proposed approach attains up to 6.19% and 9.39% improvement in the binary and multiclass classification accuracy on EyePACS. Likewise, it achieves an improvement of 2.64% on the binary classification score on APTOS with competitive multiclass classification performance. Experimental findings highlight the robustness and generalizability of the REFA‐DINO framework in classifying DR.

## 1. Introduction

Diabetic retinopathy (DR) is a common complication of diabetes and a leading cause of preventable blindness in working‐age adults. Diabetes is a chronic metabolic disorder characterized by insufficient insulin production or impaired insulin action, leading to prolonged hyperglycemia [1, 2]. According to the International Diabetes Federation, 537 million adults had diabetes worldwide in 2021, with 34%–35% developing DR and nearly 10% experiencing sight‐threatening complications [3, 4]. The rising prevalence of diabetes has substantially increased the burden of DR on healthcare systems, emphasizing the need for effective screening and early diagnosis.

DR is a progressive microvascular disease that damages retinal blood vessels because of sustained hyperglycemia. Over time, hyperglycemia weakens capillary walls, leading to microaneurysms, fluid leakage, hemorrhages, and retinal edema [5]. As the disease advances, retinal ischemia may trigger abnormal neovascularization, resulting in severe complications such as vitreous hemorrhage, retinal detachment, and irreversible vision loss if left untreated [6]. Clinically, DR is characterized by distinct retinal lesions, including microaneurysms, hemorrhages, soft and hard exudates, and neovascularization, which are key indicators for severity grading. DR is one of the most common diabetic complications and still exists in a significant number of diabetic patients all over the world [7]. However, in its early stages, DR is often asymptomatic, and its timely diagnosis is particularly challenging. As the disease progresses, patients may experience blurred vision, poor night vision, floaters, and dark regions in the visual field [8]. Early screening is therefore crucial as timely clinical interventions can significantly slow disease progression and prevent severe vision loss [9].

Existing DR classification studies initially utilized handcrafted feature extraction techniques, such as Gabor filters, morphological operators [10], histogram descriptors [11], and texture analysis, and subsequently applied machine learning (ML) classifiers [12, 13]. The sensitivity of these computationally effective methods to illumination variations, medical image context, and heterogeneous lesion appearances limits their performance in multiclass classification protocols [12]. The emergence of deep learning (DL), particularly convolutional neural networks (CNNs), enabled end‐to‐end learning directly from retinal images, significantly improving detection and classification performance [14]. Recently, vision transformers have gained popularity for modeling long‐range global retinal context using self‐attention mechanisms that are difficult for conventional CNNs to learn [15]. The advancement in DL methods further explored the hybrid CNN‐transformer models and multiscale feature learning frameworks to better capture both local lesion details and global retinal context, simultaneously [16].

Large publicly available datasets such as EyePACS, APTOS, MESSIDOR, and IDRiD have played a vital role in facilitating the development and evaluation of DL methods [17]. However, despite notable progress, existing methods struggle with class imbalance, domain shift, high computational complexity, and inconsistent performance across binary and multiclass classification [18, 19]. Furthermore, many state‐of‐the‐art (SOTA) transformer‐based and hybrid models require extensive training data with significant computational resources [20]. Robustness to low‐quality images, scalability for population‐level screening, and interpretability of model predictions remain open challenges.

To address these challenges, we introduce a relative‐efficient fusion attention framework (REFA‐DINO) that accurately identifies fundus images for binary and multiclass classification. The proposed REFA‐DINO architecture integrates a relative‐efficient fusion adapter (REFA), a cross‐attention module, and a DINOv2 transformer backbone to learn fine‐grained lesion patterns and long‐range spatial relationships simultaneously. The REFA module further comprises EfficientNet and attention‐enhanced patch embedding, enabling the learning of local and global dependencies. The cross‐attention module facilitates attention‐based interaction between convolutional and transformer representations, thereby maintaining spatial consistency across both representations. The contributions of the proposed REFA‐DINO framework are as follows:

1. Proposed a hybrid CNN‐transformer architecture, REFA‐DINO, to extract fine‐grained local features along with global dependencies for accurate DR classification.
2. Introduced a REFA module comprising EfficientNet and attention‐enhanced patch embedding, along with relative position‐aware cross‐attention to enhance alignment between local and global features.
3. Conducted extensive experiments, including cross‐corpora evaluation, ablation study, and comparative analysis utilizing two diverse datasets, to highlight the effectiveness of the proposed framework.

The rest of the paper is organized into the following sections. An overview of the relevant literature is provided in Section 2, whereas the proposed methodology is described in Section 3. Datasets, results, and discussion are presented in Section 4. Finally, Section 5 presents the conclusion and future work.

## 2. Literature Review

The most recent developments in artificial intelligence have greatly enhanced preliminary diagnosis and DR classification, as many studies used ML, DL, and hybrid methods, including transformer‐based methods. This section examines the reported SOTA ML, DL, and hybrid DR classification methods.

### 2.1. Handcrafted and Conventional ML Approaches

Traditional methods for DR classification mainly depend on handcrafted features and ML classifiers. Commonly used handcrafted techniques extracted texture, shape, and color features, which are classified through conventional ML classifiers, such as support vector machine (SVM) and random forest (RF). To enhance the discriminative capability of handcrafted retinal features, Singh et al. [18] initially obtained structural and texture‐based retinal features and optimized them through binary cuckoo search (BCS) and artificial bee colony (ABC) algorithms. The optimized feature vectors were passed to RF and SVM classifiers, which achieved an accuracy of 91.2%. Similarly, Li and Zhou [21] introduced local binary pattern and gray‐level co‐occurrence matrix descriptors and employed an RF classifier for DR classification. The method achieved an AUC of 0.89 on the APTOS‐2019 dataset, while demonstrating sensitivity to noisy images and limited robustness for multiclass DR classification.

Structural and single‐scale features are difficult to obtain, and complex structures in fundus images are often not accurately extracted at the lesion level. To address this, multiscale feature engineering and color‐based encoding techniques are used to enhance lesion‐level features. The multiscale wavelet method was introduced in [21] to extract frequency‐domain features, which were then passed to an ensemble ML classifier for DR classification. The Vornoi‐based handcrafted retinal descriptor employing the geometry‐conscious dependencies processed through an SVM achieved an AUC of 96.4% on 800 EyePACS samples [22]. In [23], a fusion of HOG and color histogram features was used with an SVM and achieved 92.1% accuracy. In summary, multiscale feature engineering methods dependent on manually designed feature representations exhibit limited scalability across diverse datasets and reduced robustness for imaging variability.

### 2.2. DL‐Based Approaches

To overcome scalability limitations, recent research has increasingly shifted from classical ML techniques to DL‐based frameworks for DR classification. In contrast to handcrafted feature‐based approaches, DL methods for image analysis automatically learn hierarchical features from retinal images. Specifically, CNNs have demonstrated a strong ability to capture lesion patterns without the need for manually designed feature descriptors. Yan et al. [24] employed a lightweight MobileNetV3 architecture and achieved 92.8% accuracy on the EyePACS dataset. Similarly, in [25], the attention‐enhanced EfficientNet was introduced for multiclass DR classification and achieved 97.4% accuracy on the APTOS‐2019 dataset. Karthik et al. [26] presented a unified lightweight CNN framework to perform binary detection and severity grading using the APTOS dataset. The method reported 96.2% accuracy for binary classification and 79.4% for severity grading; however, the model showed reduced discrimination for advanced DR stages due to subtle interclass differences. In [27], an AlexNet‐based framework was demonstrated that integrated global attention blocks for improved lesion localization and self‐convergence under severe class imbalance. Suedumrong et al. [28] employed a compact CNN combined with extensive preprocessing, including background removal and data augmentation, to perform five‐class DR classification. The approach achieved a validation accuracy of 90.6% on the EyePACS dataset, although it remained sensitive to early‐stage lesions and class imbalance. Complementarily, [29] utilized lightweight CNN backbones such as MobileNetV3 and DenseNet‐169 on the same datasets, balancing classification performance with reduced computational complexity. Mahdi et al. [30] suggested an inception‐based transfer learning model to classify OCT retinal diseases, including DME as a diabetic retinal disease, and attained 90.2% accuracy, 91.36% precision, 90.73% recall, and 90.78% F1‐score. Besides classification of the disease, Dai et al. [31] designed a retinal fundus enhancement algorithm that is based on normalized convolution, image fusion, and denoising to enhance visibility of small vessels, microaneurysms, and exudates. Muhammed [32] presented an entropy‐based optic disc localization algorithm and attained 95% accuracy on DRIVE and 89% on DIARETDBI. Deng et al. [33] proposed a vascular graph‐based retinal registration system that achieved 95.83% success rate in pairwise retinal image registration. Although the current approaches show a better performance, the DL models are still limited to the problem of generalization, lack interpretability, and high training and inference demands.

### 2.3. Hybrid and Transformer‐Based Approaches

To address the limited ability of conventional CNNs to capture long‐range dependencies and global retinal context, recent studies have increasingly explored transformer‐based frameworks for DR classification. In [34], a feature intensity vision transformer that incorporated self‐attention to learn long‐range retinal and class‐wise contextual dependencies attained an AUC of 96.8%. Nevertheless, the method was excessively data‐dependent and required a high computational cost. In [35], the residual attention vision transformer hierarchically fused tokens to obtain global retinal context. However, the model is unable to detect fine‐grained lesion details and is resistant to class imbalance and domain shift. Another method [14] used a feature extraction block (FEB) and a grading prediction block (GPB) based on a transformer for five‐stage disease classification. The model [14] employed a fine‐grained transformer and residual spatial attention to extract lesion‐level features and achieved 91.54% accuracy.

The researchers have also focused on hybrid DL techniques based on CNN and transformer architectures for DR classification. A lightweight CNN‐transformer achieved an AUC score of 0.98 on the EyePACS and APTOS datasets, indicating a high degree of discriminative ability [36]. A study [37] explored transformer‐based CNNs augmented with Grad‐CAM for interpretability, achieving 97% accuracy on the EyePACS dataset. Similarly, in [38], an explainable vision transformer model incorporating Grad‐CAM and SHAP was evaluated on the APTOS dataset, achieving 94.6% accuracy. However, integrating explainability modules introduces additional inference latency and architectural complexity. Akhtar et al. [39] presented RSG‐Net for binary and multiclass DR grading, implementing an aggressive data augmentation strategy and a contrast enhancement approach. The high reliance on dataset‐specific preprocessing and the lack of wide cross‐dataset validation limit its generalizability. In [40], an attention‐based architecture, DAM‐EfficientNet‐B0, was introduced to improve discriminative feature learning and was evaluated on the EyePACS and MESSIDOR datasets. The model depends on multistage preprocessing and auxiliary feature extractors, which may hinder scalability in real‐world clinical deployments. Transformer‐based approaches designed to extract global contextual features for complex retinal images have demonstrated reliable performance across EyePACS, APTOS, and MESSIDOR datasets. Despite this, further investigation is required for unified, efficient, and clinically deployable DR frameworks with improved generalizability and interpretability. Table 1 provides an overview of related work for DR classification.

**Table 1:** Literature review on diabetic retinopathy classification.

Year	Method	Dataset(s)	Performance	Limitations	Parameters
Handcrafted and classical machine learning methods					
2023	Machine learning algorithm (BCS, ABC) with RF and SVM [18]	Retinal fundus images	ACC: 91.2%	Reliance on handcrafted features, limited scalability	—
2025	LBP + GLCM texture descriptors with random forest [20]	APTOS‐2019	AUC: 89%	Noise sensitivity, weak multiclass robustness	—
2025	Multiscale wavelet features with ensemble ML [21]	Retinal images	AUC: 97%	High computational complexity	—
2025	Voronoi‐based geometric descriptors and SVM [22]	EyePACS (800 samples)	AUC: 96.4%	No cross‐dataset validation	—
2026	Gabor‐enhanced CNN with SMOTE [23]	MESSIDOR	ACC: ~89%–91%	Limited generalizability	—
Deep learning–based methods					
2023	MobileNetV3‐based DR classifier [24]	EyePACS	ACC: 94.0%	Limited capacity for subtle lesion discrimination	—
2026	EfficientNet‐B0 + MobileNetV1 framework [25]	EyePACS	ACC: 97.3%	High training and optimization cost	—
2025	Unified lightweight CNN for DR grading [26]	APTOS	ACC: 96.2% (binary), 79.4% (multi)	Reduced discrimination for advanced DR stages	—
2025	CNN‐based AlexNet multigrade DR model [27]	EyePACS	ACC: 95.0%	Limited interpretability	—
2024	CNN with background removal and data augmentation [28]	EyePACS	ACC: 90.6%	Sensitive to domain shift and early‐stage lesions	—
2025	Federated CNN framework with MobileNetV1 for DR detection [29]	Multicenter datasets	ACC: 95.8%	Communication overhead and synchronization complexity	—
Hybrid and transformer‐based methods					
2026	Feature Intensity Vision‐Transformer [30]	Fundus images	ACC: 96.8%	High data dependency	FLOPs: 1.5–6 G Inference time: 100–500 ms Parameters: 5–25 million
2025	Residual attention vision transformer [31]	MESSIDOR	ACC: 99.36%	Evaluated on a single dataset with homogeneous imaging	Parameters: 50 million
2023	Vision transformer with residual attention (FEB + GPB) [32]	DR fundus images	ACC: 91.5%	Dataset‐specific tuning, limited generalization	FLOPs: 10^6^ (1–2) G Inference time: 37–44 ms
2023	Compact CNN‐transformer with low‐resolution inputs [33]	EyePACS	F1‐score: 98%	Limited clinical validation	Inference time: 21.57 ms
2022	CNN‐transformer with Grad‐CAM interpretability [34]	EyePACS	ACC: 97.0%	Increased inference latency	FLOPs: 8–20 G Inference time: 100 ms Parameters: 40–55 million
2025	Explainable vision transformer with Grad‐CAM and SHAP [35]	APTOS, IDRiD	ACC: 94.6%	High computational cost due to complex model architecture	FLOPs: 4–20 G Inference time: 100–500 ms Parameters:20–90 million
2025	RSG‐Net with aggressive augmentation [14]	EyePACS	ACC: 95.0%	Heavy reliance on dataset‐specific preprocessing	—
2025	DAM‐EfficientNet‐B0 (attention‐based) [40]	EyePACS, MESSIDOR	ACC: 96.1%	Multistage preprocessing limits scalability	—

## 3. Proposed Methodology

**Figure 1:**
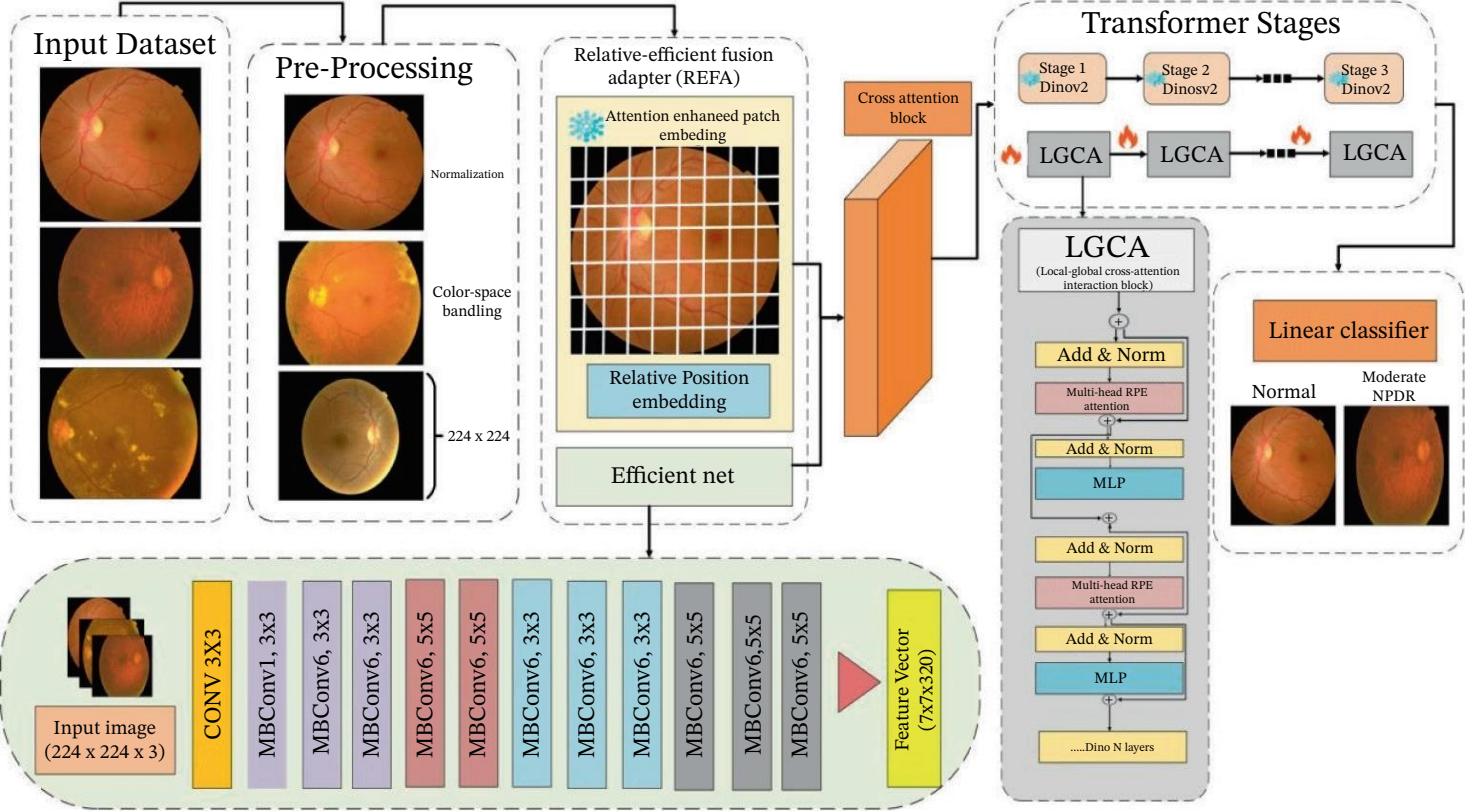
Architecture of the proposed REFA‐DINO framework.

In our proposed REFA‐DINO framework, retinal fundus images are initially resized and normalized, with color‐space operations applied to minimize illumination effects and enhance lesion visibility. The preprocessed images are passed to a REFA module with two parallel branches: an EfficientNet‐based convolutional pathway that learns local features and an attention‐enhanced patch‐embedding pathway, which learns global image representations via relative positional embeddings (RPEs). To combine local and global information, our proposed framework uses relative position‐aware cross‐attention between convolutional features and patch tokens, thereby maintaining spatial relations across the representations. The fused tokens are then refined using DINOv2 transformer stages with the local–global contextual attention (LGCA) module to learn long‐range dependencies. The LGCA module facilitates effective interaction between local lesion‐sensitive features and global contextual representations through a cross‐attention module with an MLP head, enabling the model to capture both fine‐grained pathological patterns and broader retinal structures. Lastly, a lightweight linear classifier with dropout, normalization, and a linear layer provides binary and multiclass DR classification, as shown in Figure 1.

### 3.1. Preprocessing

The input image is resized to a fixed resolution of 224 × 224 to ensure consistent input dimensions across the network. We apply intensity normalization to reduce acquisition‐dependent brightness variation and to stabilize optimization. For an input RGB fundus image *I* ∈ *ℝ* ^*H*×*W*×3^, normalization is performed channel‐wise as follows:

(1) I′=I−μσ,

where *μ* and *σ* denote the per‐channel mean and standard deviation computed from the training set. Color‐space handling is applied to reduce illumination sensitivity and improve lesion visibility. These steps reduce image‐level variability and make the model more robust to real‐world screening conditions.

### 3.2. REFA

The REFA is designed to generate complementary feature representations by jointly encoding global contextual information and local lesion‐level features in parallel. Specifically, REFA comprises an attention‐enhanced patch‐embedding branch with relative positional encoding and a parallel EfficientNet‐based local‐feature‐extraction branch. These features are then passed to a separate cross‐attention block, where local and global representations are fused via cross‐attention before being forwarded to the transformer stages.

#### 3.2.1. Local Feature Extraction Using EfficientNet

The preprocessed fundus image *I* ^′^ is forwarded through an EfficientNet backbone *f*_eff_ to extract convolutional feature maps *F* as follows:

(2) F=feffI′∈ℝh×w×c,

where *h*, *w*, and *c* represent the height, width, and channel dimensions of the feature tensor, respectively. This branch captures fine‐grained, lesion‐sensitive patterns such as microaneurysms, hemorrhages, and exudates, which are often difficult to model using global attention alone.

#### 3.2.2. Attention‐Enhanced Patch Embedding

In parallel, the preprocessed fundus image *I* ^′^ is processed through attention‐enhanced patch embedding, where it is divided into nonoverlapping patches. Each patch is linearly projected into a *D*‐dimensional embedding space, and a classification token is appended to form a sequence of patch tokens as follows in Equation (3):

(3) X=xcls;xi;⋯;xN∈ℝN+1×D,

where *x*_cls_ denotes the classification token, *x*_*i*_ represents the *i*‐th patch embedding, *N* is the total number of patches, and *D* is the embedding dimension. To emphasize early diagnostically relevant retinal regions, an attention‐enhancement mechanism is applied during patch embedding via a lightweight gating operation, as shown in Equation (4).

(4) X~=X⊙σgX,

where *X* ∈ *ℝ* ^(*N* + 1)×*D*^ denotes the original patch token sequence, including the classification token, $\tilde{X}$ represents the attention‐enhanced patch tokens, *g*(·) is a learnable transformation function implemented using a lightweight linear projection, *σ*(·) denotes the sigmoid activation function, and ⊙ represents element‐wise multiplication.

In addition, RPEs are incorporated directly into the patch tokens to preserve spatial relationships between neighboring patches, as represented in Equation (5). Unlike absolute positional encoding, RPE captures relative spatial dependencies, which are critical for modeling the structured layout of retinal anatomy and lesion distributions.

(5) X⟵X~+Erel.

The resulting token sequence *X* encodes global contextual information enriched with spatial awareness and lesion‐sensitive emphasis, which is then forwarded to the subsequent cross‐attention block along with local convolutional features.

### 3.3. Cross‐Attention Refinement Layer

Following the REFA module, a cross‐attention refinement layer is employed to strengthen the interaction between lesion‐aware local features and global contextual representations as represented in Equation (6):

(6) Xf∈ℝN+1×D,

where *X*_*f*_ denotes the fused token sequence produced after cross‐attention–based integration of the parallel representations. This refinement layer also applies self‐attention to the fused tokens to enhance contextual alignment and suppress residual inconsistencies introduced during fusion. The refined token representation $\overset{\wedge }{X}$ is computed as follows:

(7) X∧=Xf+AttnXf,Xf,Xf,

where Attn(·) represents a standard multihead attention mechanism with a residual connection. By jointly attending to the fused representations, this layer reinforces globally consistent patterns while preserving lesion‐sensitive cues.

### 3.4. Transformer Stages (DINOv2 Backbone)

After the local–global feature fusion, the obtained sequence of tokens $\overset{\wedge }{X\prime }$ is passed through a stack of transformer stages using the DINOv2 backbone. DINOv2 is used because it has a powerful ability to learn strong visual representations and to model long‐range contextual information between retinal images. Each transformer stage has a LGCA module before the basic transformer operations to further improve the discrimination of features. This design allows the network to learn both fine‐grained features of the lesions and the entire retinal scene, which are both crucial for accurate DR classification.

#### 3.4.1. LGCA

To improve the feature set of the DINOv2 backbone, an LGCA module is added to each transformer layer. The proposed LGCA uses multihead relative position encoding (RPE) attention to jointly model local lesion characteristics and global retinal structures, unlike the existing DINOv2 architecture, which is based on global self‐attention. Given the input tokens *X* at layer *l* in Equation (8), LGCA aggregates local neighborhood information and global self‐attention responses, as presented in Equation (9).

(8) Xl=x1,x2,⋯,xN,

(9) X~l=LGCAXl,

where *X* ^*l*^ denotes the token sequence at the *l*‐th transformer layer and *N* is the number of input tokens.

(10) Q=XlWQ,K=XlWK,V=XlWV,

where *W*_Q_, *W*_K_, and W_V_ are learnable projection matrices used to generate query, key, and value representations, respectively. A learnable RPE *R*_*i* *j*_ is added to the attention computation to maintain the relative positional information of patches within the retinal image. The attention score between the token *i* and token *j* is given in Equation (11):

(11) Aij=QiKjTd+Rij.

The attention weights are then normalized by the softmax function in Equation (12):

(12) αij=expAij∑m=1NexpAim.

The output of the multihead RPE attention is computed in Equation (13), where *V*_*j*_ is the value vector corresponding to the *j*‐th token.

(13) Yi=∑j=1NαijVj.

The local contextual features and the global RPE‐attention features are then combined in the LGCA module to produce refined token representations in Equation (14):

(14) X~l=LGCAXl=FusionLl,Yl.

**Figure 2:**
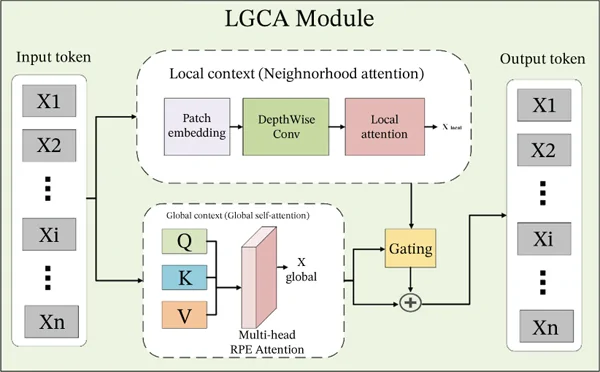
Architecture of the LGCA module.

The locally aggregated neighborhood information is *L* ^*l*^, and the global contextual features learned from the multihead RPE attention are *Y* ^*l*^. The refined tokens are then processed through a feed‐forward MLP with residual connections and layer normalization as follows:

(15) Zl=Xl+X~l 

(16) Xl+1=Zl+MLPLNZl.

where *l* denotes the index of the transformer layer. Attention is used both locally (through context aggregation) and globally (through RPE), allowing the network to effectively localize lesions and model retinal structures at various scales. Thus, the refined representations of tokens give more discriminative information for DR grading and enhance the performance of DR classification. The architecture of the LGCA module is shown in Figure 2.

### 3.5. Classification Head

After the transformer refinement stages, the final token representations are forwarded to the classification head for binary and multiclass DR prediction. The global image‐level representation is obtained from the classification token at the output of the final transformer layer, represented in Equation (17).

(17) h=XclsL,

where *L* denotes the total number of transformer layers and $X_{\text{cls}}^{L}$ represents the classification token embedding. To improve generalization and stabilize optimization, the extracted feature vector is first normalized and regularized using layer normalization, followed by dropout as follows in Equation (18):

(18) h~=DropoutLNh,

where LN(·) denotes layer normalization, which normalizes the token representations of the features, increasing numerical stability and supporting effective optimization in the classification head. The final linear classification layer that maps the learned feature representation to class logits is defined as follows:

(19) y∧=Wch~+bc,

where *W*_*c*_ and *b*_*c*_ are the learnable weights and bias parameters of the classification head. For multiclass DR grading with *K* severity levels, the model is trained using the softmax cross‐entropy loss, defined as follows:

(20) Lmulti=−∑k=1Kyklogexpy∧k∑j=1Kexpy∧j,

where *y*_*k*_ denotes the ground‐truth label for class *k*. For binary DR classification, a sigmoid activation followed by binary cross‐entropy loss is used, as mentioned in Equation (21).

(21) Lbin=−ylogσy∧+1−ylog1−σy∧,

where *σ*(·) represents the sigmoid function and *y* ∈ {0, 1} is the ground‐truth label. Collectively, the proposed framework leverages an effective fusion of local lesion‐aware features and global contextual representations, followed by a lightweight yet robust classification head, enabling accurate and scalable DR classification across both binary and multiclass settings. The entire architecture of the proposed REFA‐DINO framework is summarized in Algorithm 1.

**Algorithm 1: DR classification process of REFA‐DINO framework.**
*Input: Fundus image* *X* ∈ *ℝ* ^*H*×*W*×3^
*Output: Predicted DR class* $\overset{\wedge }{y}$ (*Binary or* 5*-class*)
*Initialize*
1: *D*⟵ *DINOv*2 *transformer stages*
2: *E*⟵ *EfficientNet-B*0
3: *P*⟵ *Attention-enhanced patch embedding*
5: *A*⟵ *Cross-attention refinement*
6: *H*⟵ *Linear classification head*
*Preprocessing*
7: *X*⟵*R* *e* *s* *i* *z* *e*(*X*, 224 × 224)
8: *X*⟵*N* *o* *r* *m* *a* *l* *i* *z* *e*(*X*)
*Relative-efficient fusion adapter (REFA) module*
9: *F*⟵*E*(*X*) *// Local feature extraction*
*// Extracts lesion-aware local retinal features*
10: *T*⟵*P*(*X*) *// Patch-level representation*
*// Generates attention-enhanced patch embeddings*
*Cross-attention refinement*
12: *C*⟵*A*(*T*, *F*)
*// Fused contextual dependencies across aligned features*
*DINOv*2 *transformer stages*
13: *D*_*f*_⟵*D*(*C*)
*// Stacked DINOv*2 *transformer stages with LGCA*
*Classification*
14: $\overset{\wedge }{y}⟵H\left(D_{f}\right)$
*// Binary or multiclass DR prediction*
15: *end for*
16: *return* $\overset{\wedge }{y}$

## 4. Experiments and Results

In this section, the details of the experimental setup and datasets utilized to assess the performance of the proposed DR classification framework are provided. This section also discusses the conducted experiments, including performance evaluation, comparison with existing methods, cross‐corpora evaluation, and ablation study, to highlight the effectiveness of the proposed REFA‐DINO framework.

### 4.1. Datasets

**Figure 3:**
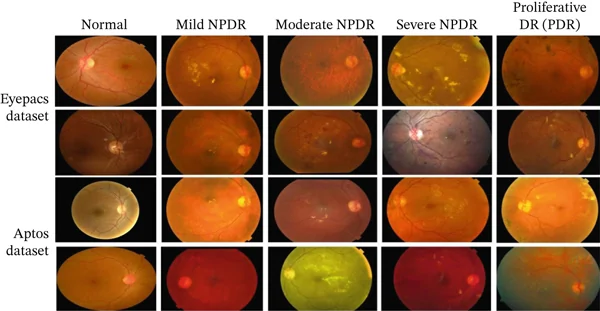
Samples of the fundus images from EyePACS and APTOS datasets.

This study used two publicly available retinal fundus image datasets, EyePACS [41] and APTOS [42]. Both datasets consist of color fundus photographs annotated by clinical experts according to the International Clinical Diabetic Retinopathy (ICDR) Severity Scale. The ICDR grading system categorizes DR into five classes: normal (no‐DR), mild NPDR, moderate NPDR, severe NPDR, and proliferative diabetic retinopathy (PDR). Some samples from both datasets are shown in Figure 3. The dataset‐specific details are provided in subsequent subsections.

#### 4.1.1. EyePACS Dataset

The EyePACS dataset is a large‐scale DR dataset collected from real‐world clinical environments and released as part of the Kaggle Diabetic Retinopathy Detection Challenge [41]. It contains high‐resolution fundus images with significant variations in illumination and image quality, closely reflecting real‐world screening conditions. EyePACS comprises 72,493 samples in the training set, 8870 in the validation set, and 9074 in the test set, while maintaining class‐wise distribution. The class‐wise distribution of image samples in the training, validation, and testing sets is provided in Table 2. The pronounced class imbalance in EyePACS poses significant challenges for multiclass DR classification.

**Table 2:** Datasets split for diabetic retinopathy classification.

Class	Training (70%)	Validation (20%)	Testing (10%)
EyePACS dataset			
Normal (no‐DR)	55,153	6710	6896
Mild NPDR	3404	419	431
Moderate NPDR	11,319	1414	1419
Severe NPDR	853	108	103
Proliferative DR (PDR)	1764	219	225
APTOS dataset			
Normal (no‐DR)	1434	172	199
Mild NPDR	1300	40	30
Moderate NPDR	808	104	87
Severe NPDR	154	22	17
Proliferative DR (PDR)	234	28	33

#### 4.1.2. APTOS Dataset

The APTOS dataset was released as part of the APTOS 2019 Blindness Detection Challenge on Kaggle [42]. It provides a smaller yet more diverse collection of retinal fundus images than the EyePACS dataset. APTOS contains 3930 for the training set, 366 for the validation set, and 366 samples for the testing set. The class‐wise DR images distribution is provided in Table 2. Although APTOS is smaller than EyePACS, notable class imbalance remains, particularly in severe NPDR and PDR categories.

### 4.2. Performance Evaluation Metrics

The performance of the proposed DR classification framework is evaluated using standard quantitative metrics commonly adopted in medical image analysis. These metrics assess the model′s reliability for both binary and multiclass DR classification tasks.

#### 4.2.1. Accuracy

Accuracy represents the proportion of correctly classified retinal fundus images among all test samples and is defined as

(22) Accuracy=TP+TNTP+TN+FP+FN,

where *T* *P* and *T* *N* denote the numbers of correctly classified DR and non‐DR images, respectively, whereas *F* *P* and *F* *N* represent false predictions.

#### 4.2.2. Balanced Accuracy

Balanced accuracy is used to evaluate classification performance under class‐imbalanced conditions. It computes the average recall obtained for each class and is defined as

(23) Balanced accuracy=1C∑i=1CTPiTPi+FNi,

where *C* denotes the total number of classes and *T* *P*_*i*_ and *F* *N*_*i*_ represent the number of true positives and false negatives for the *i*‐th class, respectively.

#### 4.2.3. Recall (Sensitivity)

Recall, also known as sensitivity, measures the model′s ability to correctly identify images belonging to a specific DR class. It is defined as

(24) Recall=TPTP+FN.

#### 4.2.4. Precision

Precision indicates the proportion of correctly predicted DR samples among all samples predicted as DR. It reflects the reliability of positive predictions and is defined as

(25) Precision=TPTP+FP.

#### 4.2.5. F1‐Score

The F1‐score provides a balanced evaluation by combining precision and recall into a single metric. It is especially useful for imbalanced multiclass DR datasets and is defined as

(26) F12score=×Recall×PrecisionRecall+Precision.

For multiclass DR classification, all metrics are computed per class and macroaveraged to ensure equal contribution from each DR severity level, including underrepresented classes such as severe NPDR and proliferative DR.

### 4.3. Experimental Setup

A hyperparameter tuning process was systematically performed to find the best training configurations by using validation performance. Numerous experiments were conducted by varying key parameters, and the selected parameters were a learning rate of 0.001, a batch size of 8, a label smoothing factor of 0.1, and a weight decay of 0.0001. The method was optimized using the Adam optimizer because its decoupled weight decay mechanism can enhance generalization and stability during training. To calculate binary DR classification, binary cross‐entropy loss with label smoothing was employed, whereas for multiclass, categorical cross‐entropy loss with label smoothing was used to mitigate overconfidence and improve resilience to noisy labels. The method is evaluated over 15 epochs and retains the best performing model checkpoints. All experiments were conducted on a high‐performance computing system with multicore processors and an RTX 3090 GPU.

### 4.4. Computational Complexity Analysis

We performed a computational complexity analysis of the proposed REFA‐DINO framework with transformer‐based and hybrid CNN‐transformer–based approaches reported in the literature. The details of the reported parameter counts, FLOPs, and inference times of the proposed and comparative models are summarized in Table 3.

**Table 3:** Computational complexity analysis of proposed and comparative models.

Method	Parameters (million)	FLOPs (G)	Inference time (ms)
Feature intensity vision transformer [30]	5–25	1.5–6	100–500
Residual attention vision transformer [31]	50	—	—
Vision transformer with residual attention (FEB + GPB) [32]	—	1‐2	37–44
Compact CNN‐transformer [33]	—	—	21.57
CNN‐transformer with Grad‐CAM [34]	40–55	8–20	100
Explainable vision transformer with Grad‐CAM and SHAP [35]	20–90	4–20	100–500
Proposed REFA‐DINO	28.42	4.76	279.52 ± 9.36

From Table 3, we observed that the transformer‐based and hybrid DR classification models differ significantly in computational demands. Although some lightweight architectures are relatively fast at inference, most transformer‐based models require a large number of parameters and incur high computational cost to obtain long‐range dependencies within retinal images. For example, in some studies, the number of parameters of the existing transformer models ranges from 20 to 90 million and FLOPs can be up to 20 G. The proposed REFA‐DINO framework provides a good balance between computational complexity and predictive performance. In particular, the model needs only 28.42 million parameters and 4.76 G FLOPs to achieve high classification accuracy, outperforming many current transformer and hybrid methods. Additionally, the mean inference time of 279.52 ± 9.36 ms indicates robust prediction performance appropriate for automated DR screening applications. These results show that the proposed REFA‐DINO framework presents a satisfactory balance between diagnostic performance and computational efficiency, supporting its potential deployment in real‐world clinical environments.

### 4.5. Performance Evaluation on EyePACS Dataset

To determine the effectiveness of the proposed method for DR binary and multiclass classification, we conducted experiments utilizing the large‐scale EyePACS dataset. For this, the proposed method is trained on the validation and training sets, whereas the testing set is used for evaluation as mentioned in Section 4.1.1. The results for class‐wise and overall accuracy, F1‐score, precision, and recall are shown in Table 4.

**Table 4:** Performance evaluation on EyePACS dataset.

Task/class	Accuracy (%)	Precision (%)	Recall/sensitivity (%)	F1‐score (%)	Specificity (%)	AUC (%)
Binary classification performance						
No‐DR	97.50	95.12	97.54	96.33	75.41	93.24
DR	75.41	86.48	75.43	85.56	97.55	92.87
Overall binary performance	93.77	90.70	86.48	88.40	86.48	94.50
Multiclassification performance						
Normal (no‐DR)	97.27	91.24	97.27	94.16	70.43	96.18
Mild NPDR	9.05	44.83	9.05	15.06	99.44	62.34
Moderate NPDR	72.94	73.04	72.94	72.99	95.01	87.46
Severe NPDR	14.56	44.124	14.56	21.90	99.79	65.28
Proliferative DR (PDR)	64.00	78.26	64.00	70.42	99.55	84.39
Overall multiclass performance	87.51	85.33	87.51	85.68	92.84	79.13

For the binary classification task, REFA‐DINO achieved an accuracy of 93.77% on the EyePACS dataset. The model demonstrated strong discrimination between no‐DR and DR cases, with high precision and recall values, indicating reliable learning of disease‐related retinal patterns despite large intraclass variability. In the multiclass DR grading setting, the proposed framework achieved an overall accuracy of 87.51%. High performance was observed for the no‐DR, moderate NPDR, and PDR classes, reflecting the model′s ability to capture clinically significant lesion patterns and global retinal context. In contrast, mild NPDR and severe NPDR achieved lower accuracies of 9.05% and 14.56%, respectively. Mild NPDR showed a precision of 44.83%, a recall of 9.05%, and an F1‐score of 15.06%, indicating that although some predictions are correct, most samples are missed and frequently misclassified as no‐DR and moderate NPDR due to subtle visual differences and class dominance. Similarly, severe NPDR attained a precision of 44.12%, a recall of 14.56%, and an F1‐score of 21.90%, reflecting frequent confusion with moderate NPDR and PDR due to overlapping lesion patterns, leading to increased interclass confusion. These results confirm that reduced performance primarily arises from severe class imbalance and high interclass visual similarity. Specifically, the samples from minority classes (mild NPDR and severe NPDR) are misclassified to clinically adjacent disease stages and visually similar majority classes, including moderate NPDR, no‐DR, and PDR.

**Figure 4:**
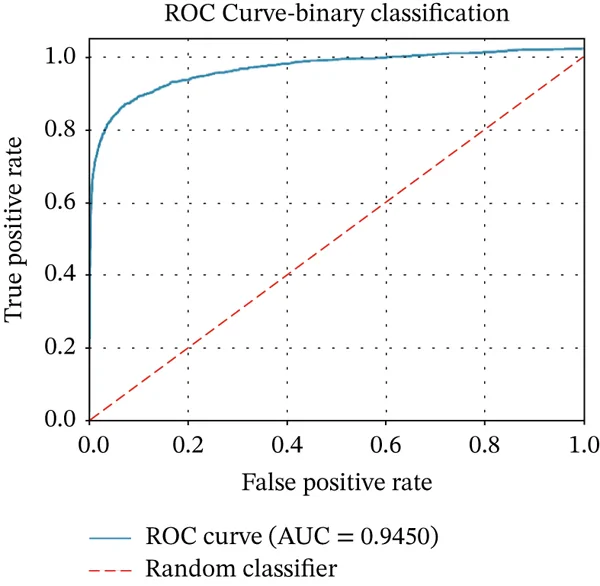
ROC curve for binary classification on EyePACS dataset.

The performance of the proposed framework (REFA‐DINO) was further analyzed using ROC‐AUC, precision, recall, and F1‐score metrics as summarized in Table 4. The proposed framework achieved an AUC of 94.50% for binary classification, as illustrated in Figure 4, which demonstrates good discriminative ability of the proposed framework and its effectiveness in distinguishing between no‐DR and DR cases. In the multiclass scenario, the class‐wise precision, recall, and F1‐score results suggest good classification accuracy across various DR severity classes. However, relatively lower recall values for minority classes indicate that there are some samples that are misclassified into visually similar neighboring classes. This is expected behavior because of class imbalance and the fact that there are subtle differences in the visual data in adjacent DR grades.

**Figure 5:**
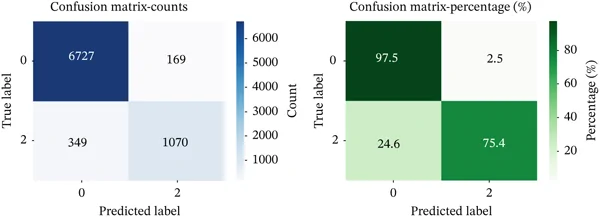
Confusion matrix for binary classification on EyePACS dataset.

**Figure 6:**
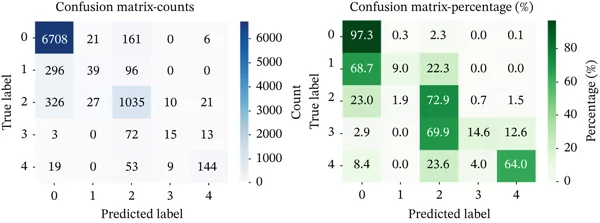
Confusion matrix for multiclassification on EyePACS dataset.

The confusion matrices for binary classification of DR and multiclass classification of DR are shown in Figures 5 and 6, respectively. In binary classification, the proposed model has achieved a better classification accuracy with a few misclassification scenarios. Likewise, the multiclass confusion matrix suggests that classification results are good at each severity level and there are only a few misclassified instances. In general, the errors observed are mainly within the DR classes that are visually close to each other, due to the severe class imbalance and high intraclass visual similarity. In conclusion, the confusion matrix analysis for both binary and multiclass DR classification further supports the robustness and effectiveness of the proposed REFA‐DINO framework.

Collectively, these findings demonstrate that REFA‐DINO effectively identifies dominant DR stages and maintains stable performance under class‐biased conditions. The integration of lesion‐aware convolutional features, attention‐enhanced patch embeddings with positional encoding, and transformer‐based global‐context modeling improves robustness toward the large, heterogeneous retinal datasets such as EyePACS.

### 4.6. Performance Evaluation on APTOS Dataset

This experiment aimed to evaluate the robustness of the proposed REFA‐DINO framework on a different, diverse dataset with varied imaging characteristics. Experiments are conducted on the APTOS dataset for both binary and multiclass classification settings, and the results are reported in Table 5. APTOS, being smaller in size, presents real‐world clinical issues such as inconsistent illumination and resolution variation, making it difficult to make predictions of heterogeneous samples. Despite that, the framework attained 98.60% accuracy for binary classification, highlighting a robust discrimination between no‐DR and DR cases. Moreover, the high precision and recall values for both classes indicate stable decision boundaries and minimal bias toward either class, confirming the robustness of the proposed framework for binary DR screening tasks. For the multiclass DR grading, the REFA‐DINO framework achieved 85.52% accuracy. The model performed well on the no‐DR, moderate NPDR, and PDR classes, which benefit from relatively higher sample availability and more distinctive pathological patterns. In contrast, the mild NPDR and severe NPDR classes exhibited lower classification accuracy, mainly due to the following two primary factors. First, these classes suffer from severe class imbalance, as shown in Table 2, resulting in limited representative samples during training. Second, mild NPDR shares subtle visual characteristics with no‐DR images, whereas severe NPDR exhibits overlapping lesion patterns with moderate NPDR, thus increasing interclass ambiguity. Several independent runs were performed to assess model stability for various initializations and shuffling of the data. The proposed method achieved an accuracy of 97.61*%* ± 0.84*%* for binary and 84.32*%* ± 1.09*%* for multiclass DR classification, demonstrating consistent and robust performance. This observation is further supported by the precision–recall trends, where higher precision but lower recall indicates that samples from these minority classes are often misclassified into visually adjacent majority classes rather than being randomly confused.

**Table 5:** Performance evaluation on APTOS dataset.

Task/class	Accuracy (%)	Precision (%)	Recall/sensitivity (%)	F1‐score (%)	Specificity (%)	AUC (%)
Binary classification performance						
No‐DR	99.42	99.38	99.86	99.62	96.55	99.21
DR	99.04	99.75	98.89	99.32	99.50	99.08
Overall binary performance	98.60	99.56	99.37	99.47	98.03	99.14
Multiclass performance						
Normal (no‐DR)	99.50	98.51	99.50	99.00	98.20	99.32
Mild NPDR	22.33	63.64	23.33	34.15	98.81	67.45
Moderate NPDR	95.40	68.03	95.40	79.43	86.02	91.84
Severe NPDR	23.53	57.14	23.53	33.33	99.14	68.27
Proliferative DR (PDR)	63.64	84.00	63.64	72.41	98.80	85.91
Overall multiclass performance	85.52	74.26	61.07	63.66	96.19	82.56

**Figure 7:**
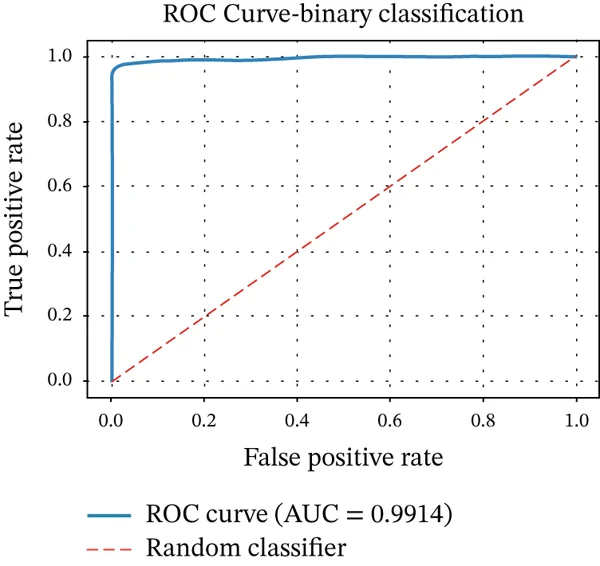
ROC curve for binary classification on APTOS dataset.

The proposed REFA‐DINO framework was evaluated in terms of AUC, precision, recall, and F1‐score as shown in Table 5. The ROC curve for binary classification is presented in Figure 7, where the AUC of 99.14% confirms the satisfactory discriminative performance of the proposed framework and the effective separation between the no‐DR and DR classes. Moreover, high precision and recall scores for both classes suggest that the classification boundaries are stable and not biased against either class, which further demonstrates the effectiveness of the proposed binary DR screening method.

**Figure 8:**
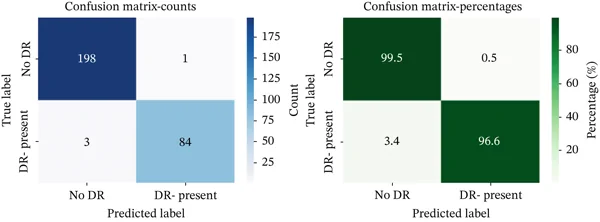
Confusion matrix for binary classification on APTOS dataset.

**Figure 9:**
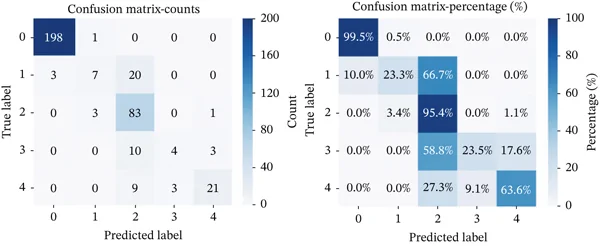
Confusion matrix for the multiclassification on APTOS dataset.

The model′s discriminative performance across different DR severity levels under imbalanced conditions is also validated by the class‐wise AUC reported in Table 5 for multiclass DR classification. The confusion matrices for binary and multiclassification are presented in Figures 8 and 9, respectively, and reveal that the class imbalance and high intraclass visual similarity are the main reasons for the lower performance of the model. In particular, samples from minority classes (mild NPDR and severe NPDR) are predominantly misclassified as majority classes (moderate NPDR, no‐DR, and PDR), which are visually similar and clinically adjacent. The results show the potential of the proposed framework to learn discriminative retinal features and emphasize the difficulty of fine‐grained multiclass DR classification.

Collectively, these results demonstrate that the REFA‐DINO framework can effectively handle nonhomogeneous class distributions and real‐world imaging variability. The proposed REFA module and cross‐attention mechanism enable the model to learn discriminative representations even under severe imbalance, making it suitable for practical screening scenarios.

### 4.7. Ablation Study

To analyze the contribution of individual architectural components in the proposed REFA‐DINO framework, we conducted an ablation study focusing on the impact of feature extraction, attention mechanisms, and fusion strategy on classification performance. The objective of this experiment was to evaluate the effectiveness of our method for progressive architectural modifications and to quantify the performance gains introduced by each modification. The ablation experiments were performed on the binary EyePACS dataset. In particular, we evaluated a series of incremental configurations, starting with convolutional feature extraction alone, then adding attention patch embedding to increase feature visibility in DR classification. In DINO architecture, we first experimented using the original MHSA module of the standard DINOv2 model. Next, an experiment was conducted by adding cross‐attention to the MHSA module, named LGCA, which improved the accuracy by 2% in the proposed framework, and finally incorporating the proposed REFA module and cross‐attention–based feature alignment. The quantitative results of these experiments are summarized in Table 6.

**Table 6:** Ablation study utilizing binary EyePACS dataset.

Method variations	Accuracy (%)	No‐DR (%)	DR (%)
Pretrained DINOv2 base	74.31	75	73
DINOv2 + EfficientNet‐B0	85.17	85	85
DINOv2 + EfficientNet‐B0 + attention patch embedding	85.69	86	86
DINOv2 (remove the cross‐attention in LGCA module) + EfficientNet‐B0 + attention‐enhanced patch embedding	85.93	86	85
DINOv2 (LGCA with the cross‐attention) + REFA module (EfficientNet‐B0 + attention‐enhanced patch embedding)	87.24	88	87
REFA‐DINO (DINOv2 with LGCA + REFA module + cross‐attention)	93.77	97.50	75.41

The findings demonstrate a consistent improvement in classification accuracy with each added module, confirming that the REFA module, comprising EfficientNet‐B0 and attention‐enhanced patch embedding, contributes positively to performance enhancement. In particular, the complete REFA‐DINO configuration achieved the highest accuracy, highlighting the effectiveness of coordinated local–global feature interaction in improving binary DR discrimination. Collectively, the ablation results indicate that each architectural improvement is positively correlated with performance gains, underscoring the effectiveness of the proposed REFA‐DINO framework.

### 4.8. Comparison With SOTA Methods

In this section, we compared the performance of the proposed REFA‐DINO framework with several SOTA CNN‐based, transformer‐based, and hybrid models for binary and multiclass DR classification, utilizing EyePACS and APTOS datasets. All the models were evaluated using the same experimental protocols mentioned in Sections 4.1.1 and 4.1.2, to ensure a fair comparison, and the corresponding results are reported in Table 7.

**Table 7:** Comparison with state‐of‐the‐art methods on EyePACS and APTOS datasets for binary and multiclass DR classification.

Methods	EyePACS		APTOS	
	Acc. (%)	Balanced acc. (%)	Acc. (%)	Balanced acc. (%)
Binary classification				
RSGNET [39]	87.58	52.00	94.41	92.42
TAHDL [26]	82.93	50.00	92.75	91.72
Custom‐CNN [28]	82.93	50.00	92.21	93.84
DAM‐EfficientNet‐B0 [40]	82.93	50.00	99.64	99.71
CNN [27]	82.59	50.00	90.21	87.14
REFA‐DINO (proposed)	93.77	86.48	98.60	97.21
Multiclass classification				
RSGNET [39]	79.63	42.17	75.96	41.74
TAHDL [26]	61.12	24.13	74.04	37.46
Custom‐CNN [28]	80.25	25.95	74.04	37.07
DAM‐EfficientNet‐B0 [40]	72.68	36.70	83.33	61.13
CNN [27]	76.96	42.16	72.68	36.70
REFA‐DINO (proposed)	87.51	51.56	85.52	61.08

For binary DR classification, existing methods showed better performance on the APTOS dataset, with an accuracy in the range of 90%–94%, whereas a performance drop was observed on the EyePACS dataset, with an accuracy in the range of 82%–84%. However, the proposed framework achieved the best performance across both datasets compared with current SOTA models. Particularly, the proposed REFA‐DINO has consistently improved overall accuracy by 6% on EyePACS and 8% on APTOS, for the binary classification task. In the more challenging multiclass DR grading task, REFA‐DINO consistently outperformed all competing methods on both datasets, achieving 87.51% accuracy on EyePACS and 85.52% on APTOS. Existing CNN‐based and hybrid methods showed inconsistent performance, particularly on the EyePACS dataset, where severe class imbalance and overlapping DR stages degrade class separability. These models often overfit dominant classes and fail to generalize under complex visual variations. In contrast, REFA‐DINO demonstrates more stable performance with an accuracy gain of 8% and 10% on EyePACS and APTOS, respectively.

Collectively, the comparative results indicate that although existing CNN‐based and hybrid models perform adequately in controlled or binary classification scenarios, their effectiveness degrades in multiclass evaluations. Conversely, REFA‐DINO demonstrates stable and competitive performance across both binary and multiclass classification tasks, even on datasets with limited samples and higher variability. The comparative study validates that the proposed framework achieves superior and more consistent performance than SOTA methods, highlighting its effectiveness in learning robust and discriminative representations for DR classification.

### 4.9. Comparison With DL Models

In this section, we compared the performance of various DL models for binary DR classification against the proposed REFA‐DINO framework. All experiments were conducted under the same evaluation protocol mentioned in Section 4.1.1, using the binary EyePACS dataset. The comparative results are reported in Table 8.

**Table 8:** Comparison with deep learning methods on the EyePACS dataset (binary DR classification).

Method	Accuracy (%)	No‐DR (%)	DR (%)
EfficientNet [40]	85	86	84
CNN [43]	79	70	67
DINOv2‐small [44]	67	65	69
DINOv2‐base [44]	74	75	73
ConvNeXt [45]	87	87	87
MedViT [46]	75	77	73
REFA‐DINO	98.60	99.04	99.42

It can be observed that conventional CNNs, pure transformer backbones, and existing hybrid models achieved moderate performance due to their limited ability to jointly capture fine‐grained lesion details and global retinal context. Conventional CNN models [43] achieved lower accuracy for both no‐DR (70%) and DR (67%) classes, indicating weak lesion sensitivity. EfficientNet [40] improved class‐wise accuracy with 86% for no‐DR and 84% for DR, but still showed performance gaps. Pure transformer backbones [44] (DINOv2‐small and DINOv2‐base) showed reduced class‐wise accuracy, particularly in no‐DR cases, suggesting difficulty in learning fine‐grained lesion cues with global token representations alone. Hybrid models such as ConvNeXt [45] and MedViT [46] provided more balanced class‐wise performance; however, their discrimination remained suboptimal. In contrast, the proposed REFA‐DINO framework achieved the highest overall accuracy of 98.60% and consistently high class‐wise accuracies (99.04% for no‐DR and 99.42% for DR), demonstrating superior robustness and balanced discrimination across both classes. Generally, existing DL models struggle to achieve balanced discrimination between no‐DR and DR classes, highlighting their reduced effectiveness compared with the proposed REFA‐DINO framework. This highlights the effectiveness of jointly combining local lesion‐sensitive convolutional features and a global transformer representation via attention‐directed alignment. All these findings suggest that REFA‐DINO is more robust and discriminative for binary DR classification.

### 4.10. Cross‐Corpora Evaluation

The cross‐corpora evaluation was also conducted to assess the generalizability of our proposed method for multiclass DR classification, utilizing APTOS and EyePACS datasets. The primary objective of cross‐corpora assessment is to evaluate the practical applicability of the REFA‐DINO framework. In particular, the method is trained on all classes of one dataset and directly tested on the other dataset, without any domain‐specific fine‐tuning, thereby simulating a realistic deployment scenario. Table 9 indicates that the proposed model trained on the EyePACS dataset achieved an accuracy of 85.52% when tested on the APTOS dataset. On the other hand, the REFA‐DINO trained on APTOS and tested on EyePACS attained an accuracy of 77.18%. It can be observed that the proposed method achieved comparatively better performance on the APTOS dataset in the cross‐corpora setting. This is mainly due to the larger sample size of EyePACS dataset, which enables the proposed model to learn more comprehensive and discriminative retinal representations during training, thereby improving generalization on the APTOS test set. In contrast, the moderate performance drop occurred in the case of EyePACS dataset. This can be attributed to the lack of imaging variation and training samples in the APTOS dataset, which limits the proposed framework′s exposure to various pathological appearances during training. In general, the cross‐corpora findings validate that the proposed REFA‐DINO has stable generalization ability and solid pathological pattern recognition in the case of heterogeneous retinal data, thus, demonstrating the framework′s ability to capture domain‐insensitive lesion‐level and global contextual features.

**Table 9:** Cross‐corpora evaluation (multiclass DR classification).

Training dataset	Testing dataset	Test accuracy
EyePACS	APTOS	85.52%
APTOS	EyePACS	77.18%

### 4.11. Discussion

It is important to develop a strong, generalizable automated DR classification system across diverse datasets and cross‐domain settings to enable large‐scale screening and timely clinical intervention. In practice, DR screening systems must handle substantial variation in image quality, acquisition equipment, disease severity distribution, and severe class imbalance without compromising diagnostic quality. Although recent CNN, transformer‐based, and hybrid CNN‐transformer methods have shown encouraging outcomes, most existing approaches are limited by optimization to a single dataset, prone to domain shift, computationally expensive, and less robust in practical clinical settings. These problems underscore the need for an efficient and flexible architecture that can adequately capture fine‐grained lesion patterns and global retinal context without sacrificing feasibility in resource‐constrained clinical and population‐level screening systems. In this respect, the proposed REFA‐DINO framework will help overcome these drawbacks by providing an effective interplay between local and global representations within a single, scalable learning framework.

The introduced REFA‐DINO architecture incorporates a REFA, consisting of an EfficientNet backbone that extracts local, lesion‐sensitive features and an attention‐enhanced patch‐embedding component with relative positional encoding to model global retinal context. These two branches are then aligned and integrated through a cross‐attention mechanism for feature fusion. Transformer stages are then applied to the fused representations, and ultimately, the resulting representations are passed to a linear classification head for binary and multiclass DR classification.

The usefulness of the proposed REFA‐DINO framework was evaluated through extensive experiments on both the EyePACS and APTOS datasets. For binary classification, the framework demonstrates strong discriminative ability between DR and no‐DR cases under heterogeneous imaging conditions, with an accuracy of 93.77% and 98.60% on EyePACS and APTOS datasets, respectively. The multiclass DR grading is more challenging than binary DR detection because of the presence of minor interclass differences and extreme class imbalance between disease stages. However, REFA‐DINO achieved notable accuracies on both EyePACS (87.51%) and APTOS (85.52%) for multiclass DR classification, as shown in Tables 4 and 5. Moreover, the superiority of the proposed framework is further highlighted through the comparison against SOTA CNN, transformer, and hybrid‐based architectures, under similar experimental settings.

The ablation study further validates the effectiveness of the proposed architecture, showing that performance improves gradually with each architectural component. The progressive integration of EfficientNet‐based local feature extraction and attention‐enhanced patch embedding leads to incremental accuracy gains, with the complete REFA‐DINO framework achieving the highest performance on the binary classification task, as mentioned in Table 6. In addition, the cross‐corpora evaluation results indicate that REFA‐DINO maintains stable performance across diverse datasets, highlighting its robustness to changes in data distribution, imaging quality, and disease prevalence.

Generally, the proposed framework improves discrimination among visually similar DR stages, even under challenging conditions such as class imbalance and heterogeneous image quality. Although the current results are promising, future extensions may include lesion‐level explainability, integration of multimodal clinical metadata, and validation in real‐world screening workflows. Nevertheless, the experimental findings confirm that REFA‐DINO is a reliable, scalable, and effective solution for automated DR classification, capable of supporting both binary detection and multiclass severity grading across diverse clinical imaging scenarios.

## 5. Conclusion

This paper has presented a novel and robust hybrid DL framework, REFA‐DINO, for binary and multi‐DR classification of retinal fundus images. Extensive experiments on the EyePACS and APTOS datasets show that REFA‐DINO consistently outperforms several SOTA CNN, transformer, and hybrid approaches under similar experimental settings. The proposed REFA‐DINO framework achieves better performance on the EyePACS dataset and shows good generalization performance on the APTOS dataset. It has performed consistently better in both binary and multiclass classification, which represents the effectiveness of feature fusion and attention mechanisms in the DR grading problem. Moreover, ablation study and cross‐dataset evaluation further confirm the framework′s effectiveness and generalization ability, robustness to class imbalance, and stability across varying imaging conditions, all of which are critical for real‐world screening scenarios. The experimental results show that REFA‐DINO is a reliable and scalable approach to DR screening in large datasets; however, performance is relatively low on minority classes, suggesting that the issue of class imbalance is still a challenge for fine‐grained severity assessment. In future work, the proposed framework can be extended to a unified architecture for segmentation and classification, enabling the simultaneous classification of lesions and the assessment of DR severity within a single end‐to‐end framework. Additionally, incorporating multimodal clinical data and validating it in real‐world screening environments can further enhance its clinical applicability.

## Funding

No funding was received for this manuscript. Open access publishing facilitated by Aalto‐yliopisto, as part of the Wiley ‐ FinELib agreement.

## Conflicts of Interest

The authors declare no conflicts of interest.

## Data Availability

We have used two publicly available datasets, that is, EyePACS and APTOS, which can be found at [43] and [44] in the paper.
